# Acupuncture Alters Brain's Dynamic Functional Network Connectivity in Stroke Patients with Motor Dysfunction: A Randomised Controlled Neuroimaging Trial

**DOI:** 10.1155/2023/8510213

**Published:** 2023-06-20

**Authors:** Yahui Wang, Mengxin Lu, Ruoyi Liu, Liping Wang, Yue Wang, Lingling Xu, Kang Wu, Chen Chen, Tianzhu Chen, Xinyue Shi, Kuangshi Li, Yihuai Zou

**Affiliations:** ^1^Department of Neurology, Dongzhimen Hospital, Beijing University of Chinese Medicine, Beijing, China; ^2^Department of Rehabilitation Medicine, Beijing Tsinghua Changgung Hospital, School of Clinical Medicine, Tsinghua University, Beijing, China; ^3^China-Japan Friendship Hospital, Beijing, China

## Abstract

**Objectives:**

Neuroimaging studies have confirmed that acupuncture can promote static functional reorganization in poststroke patients with motor dysfunction. But its effect on dynamic brain networks remains unclear. This study is aimed at investigating how acupuncture affected the brain's dynamic functional network connectivity (dFNC) after ischemic stroke.

**Methods:**

We conducted a single-center, randomised controlled neuroimaging study in ischemic stroke patients. A total of 53 patients were randomly divided into the true acupoint treatment group (TATG) and the sham acupoint treatment group (SATG) at a ratio of 2 : 1. Clinical assessments and magnetic resonance imaging (MRI) scans were performed on subjects before and after treatment. We used dFNC analysis to estimate distinct dynamic connectivity states. Then, the temporal properties and strength of functional connectivity (FC) matrix were compared within and between the two groups. The correlation analysis between dynamic characteristics and clinical scales was also calculated.

**Results:**

All functional network connectivity (FNC) matrices were clustered into 3 connectivity states. After treatment, the TATG group showed a reduced mean dwell time and found attenuated FC between the sensorimotor network (SMN) and the frontoparietal network (FPN) in state 3, which was a sparsely connected state. The FC between the dorsal attention network (DAN) and the default mode network (DMN) was higher after treatment in the TATG group in state 1, which was a relative segregated state. The SATG group preferred to increase the mean dwell time and FC within FPN in state 2, which displayed a local tightly connected state. In addition, we found that the FC value increased between DAN and right frontoparietal network (RFPN) in state 1 in the TATG group after treatment compared to the SATG group. Correlation analyses before treatment showed that the Fugl-Meyer Assessment (FMA) lower score was negatively correlated with the mean dwell time in state 3. FMA score showed positive correlation with FC in RFPN-SMN in state 3. FMA-lower score was positively correlated with FC in DAN-DMN and DAN-RFPN in state 1.

**Conclusions:**

Acupuncture has the potential to modulate abnormal temporal properties and promote the balance of separation and integration of brain function. True acupoint stimulation may have a more positive effect on regulating the brain's dynamic function. *Clinical Trial Registration*. This trial is registered with Chinese Clinical Trials Registry (ChiCTR1800016263).

## 1. Introduction

Stroke remains the second leading cause of death and the third leading cause of disability worldwide, according to the latest Global Burden of Disease study [1]. In China, ischemic stroke is the leading cause of mortality and disability, resulting in an increasing annual disease burden [2]. Motor dysfunction is the most common symptom of ischemic stroke, with more than 70% of stroke survivors suffering from a varying degree of motor dysfunction [3]. Rehabilitation services, which promote spontaneous neurological recovery and experience-dependent plasticity, are the primary approaches for promoting functional recovery and independence of patients with stroke [4–6].

Acupuncture, as a vital part of Traditional Chinese Medicine, has gained increasing acceptance worldwide and has been applied in different forms to treat diseases in more than 180 countries around the world [7–10]. In China, acupuncture is widely used in the treatment of stroke patients with motor dysfunction [11]. American Heart Association/American Stroke Association Guideline also indicated that facilitating acupuncture may be effective as an adjunctive treatment for motor recovery and walking mobility [6]. Of note, *Shou Zu Shi Er Zhen* is the representative needling prescription in China for the treatment of poststroke patients with motor dysfunction, and its effectiveness has been identified by several randomised clinical trials [12–15]. However, its underlying mechanism remains unclear.

As a noninvasive neuroimaging technology, functional magnetic resonance imaging (fMRI) lays the foundation for real-time measurement of whole-brain activity and helps advance the understanding of neuroplasticity modulated by acupuncture [16, 17]. Neuroimaging evidence has demonstrated the facilitating effect of acupuncture in the regulation of brain functional reorganization, comprising activation of motor-related cortical areas [18–20], increasing motor-cognition connectivity and decreasing compensation of contralesional motor cortex, and modulating effective connectivity of resting-state networks in patients with poststroke hemiplegia [21, 22]. However, these studies mainly focus on instant acupuncture effects. Our previous work provided evidence for long-term acupuncture intervention effects using the voxel-mirrored homotopic connectivity method. We confirmed that *Shou Zu Shi Er Zhen* acupuncture prescription can integrate information among motor, vision, hearing processing, and cognitive brain regions, thereby promoting motor function recovery [23].

Despite such progress, the assessment of brain function is still limited by an assumption that the brain activity is constant throughout recording periods in resting state [24]. Recent studies have confirmed the time-varying properties of functional brain networks [25, 26]. Dynamic functional network connectivity (dFNC), which combines independent component analysis and sliding window approach, reflects temporal variations in functional network connectivity at much faster timescales (seconds-minutes) [24, 25]. Several studies have confirmed that acupuncture can affect the brain's dynamic functional connectivity (FC), such as decreasing temporal variability in chronic tinnitus and regulating dynamic alterations of brain activity in migraine patients [27, 28]. So far, no study has shown the acupuncture effect on dFNC in stroke patients. However, there is evidence suggesting that the brain's preference for distinct dynamic connection patterns was altered after ischemic stroke, and the specific patterns may be critical for neurological function recovery [29].

Therefore, the goal of the present study was to investigate how acupuncture affected the brain's dynamic connections following ischemic stroke based on dFNC analysis. We hypothesized that acupuncture might modulate dynamic properties of brain functional networks in poststroke patients, while true acupoint stimulation has a more positive effect.

## 2. Materials and Methods

### 2.1. Study Design

This was a single-center, randomised controlled neuroimaging study, with patients and assessors blinded for group allocation. According to the order of admission, the stroke patients were randomly divided into the true acupoint treatment group (TATG) and the sham acupoint treatment group (SATG) at a ratio of 2 : 1 using a random number table. Based on conventional treatment, the TATG group received *Shou Zu Shi Er Zhen* acupuncture treatment, while the SATG group received sham acupuncture treatment. All patients were evaluated using clinical scales and underwent MRI scan before and after acupuncture treatment. The trial was registered in the Chinese Clinical Trial Registry (Registration number: ChiCTR 1800016263). The study protocol had been published previously [30].

### 2.2. Participants

**Figure 1:**
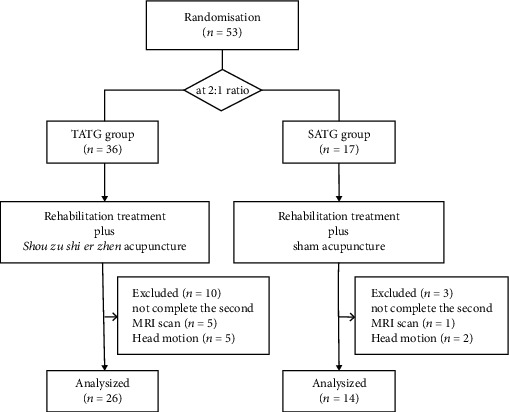
The study flow chart.

A total of 53 patients who matched the selection criteria were recruited from Dongzhimen Hospital affiliated to Beijing University of Chinese Medicine (Beijing, China) from March 2018 to December 2021. The inclusion criteria were as follows: (1) patients with ischemic stroke; (2) within 6 weeks from onset; (3) single lesion of ischemic infarct restricted to the unilateral hemisphere involving the internal capsule, basal ganglia, corona radiata, and its neighboring regions; (4) right-handed before stroke; (5) 35-80 years of age; (6) consciousness and stable condition; (7) not taken any psychiatric medications in 1 month. The exclusion criteria were as follows: (1) severe primary diseases of heart, liver, kidneys, hematologic system, immune system disease, tumors, and psychiatric disorders; (2) pregnant or lactating women; (3) any MRI contraindications; (4) any brain abnormalities except infarction identified by MRI. Seven patients did not complete the second MRI scan for personal reasons, and six patients were excluded due to severe head motion during scanning. Therefore, a total of 40 patients were included in the final analysis.  Figure 1 shows the flow chart of this study.

### 2.3. Interventions

Ande brand disposable acupuncture needles (size 0.25 × 40 mm) were used. Patients in the TATG group received *Shou Zu Shi Er Zhen* acupuncture treatment, which consist of bilateral Hegu (LI4), Quchi (LI11), Neiguan (PC6), Zusanli (ST36), Yanglingquan (GB34), and Sanyinjiao (SP6). After skin disinfection, acupuncture needles were inserted vertically into the skin to a depth of 20-30 mm. Following needle insertion, acupuncture manipulations of twirling and lifting were conducted on all needles to allow patients to obtain *De qi*, a combination of sensations including soreness, dullness, numbness, heaviness, and other feelings [31]. It was believed to play an important role in acupuncture efficacy. Needles were kept in situ for 30 minutes. The patients received five treatment sessions per week for 2 weeks (10 sessions in total). Patients in the SATG group received sham acupuncture treatment. The location of sham acupoints was referred to the method published in JAMA by Liu et al., with 20 mm beside the true acupoints [32]. The location of all acupoints is shown in Supplementary Material 1. We would avoid *De qi* sensations in the manipulation in the SATG group. Besides that, other treatment procedures were the same as the TATG group. And all the patients received routine medication and rehabilitation treatment.

### 2.4. Clinical Assessments

We used the National Institutes of Health Stroke Scale (NIHSS) to assess the severity of neurological deficit and the Fugl-Meyer Assessment (FMA) to evaluate the motor function at baseline and after the acupuncture treatment. SPSS 20 was applied for statistical analysis. Paired samples *t*-test was used for within-group comparisons. Independent sample *t*-test was used to compare the difference between the two groups. Statistical significance was set at *P* < 0.05.

### 2.5. MRI Data Acquisition

All MRI data were acquired from a 3.0 Tesla scanner (Siemens, Sonata, Germany) at Dongzhimen Hospital, Beijing, China. The parameters of gradient echoplanar imaging (EPI) were as follows: echo time (TE) = 30 ms, repetition time (TR) = 2000 ms, matrix = 64 × 64, field of vision (FOV) = 225 mm × 225 mm, slice thickness = 3.5 mm, and flip angle (FA) = 90°. The functional scan lasted for 6 minutes, and 179 image volumes were obtained. And the T1-weighted imaging (T1WI) scan lasted for 4 minutes and 10 seconds, with the following parameters: TE = 2.53 ms, TR = 1900 ms, matrix = 256 × 256, FOV = 250 mm × 250 mm, slice thickness = 1.0 mm, and FA = 9°.

### 2.6. Preprocessing of fMRI Data

The resting-state fMRI data were preprocessed using DPABI software [33] in MATLAB (version R2018a, MathWorks, Inc., Natick, MA, USA). For the left hemispheric lesions (*n* = 18), the functional images were flipped along the midsagittal plane in order to reduce the spatial heterogeneity of lesions. Thus, all lesions were located on the right hemisphere. The probability map and detailed information of the lesion location in each patient are shown in Supplementary Material 1. The first 10 time points were removed to stabilize MRI signal and remained 169 volumes. Then, the remaining volumes were slice-timing corrected to correct time differences between slices. Head motion correction was performed, and the patients were excluded when the maximum translation exceeded 3 mm and rotational parameters exceeded 3 degrees in any direction. Next functional images were normalized to the standard Montreal Neurological Institute space by using T1 image unified segmentation, resliced into 3 × 3 × 3 mm^3^. Afterward, images were smoothed using a Gaussian kernel with a 4 mm full width at half maximum, regressed with 24-parameter regression, white matter, and cerebrospinal fluid signal. Finally, the data were bandpass filtered with 0.01-0.1 Hz. Global signal regression was not performed.

### 2.7. Intrinsic Connectivity Networks

For all preprocessing data, spatial independent component analysis (ICA) was carried out to identify intrinsic connectivity network independent components (ICs) using the Group ICA Toolbox (GIFT version 4.0b, http://icatb.sourceforge.net). Firstly, the number of ICs was automatically estimated from the imaging data of all the patient, and 56 ICs were obtained. Following that, the principal component analysis was performed to reduce dimensionality. Secondly, Group ICA was conducted using the infomax algorithm to extract independent spatial maps and time courses of each component [34]. Lastly, the back-reconstruction [35] and Fisher's *z*-transformation were performed for each independent component of the subjects so that the transformed data approximately obeyed a normal distribution with standard deviation of the mean. Multiple regression analysis was applied to analyze the spatial correlation between the extracted components and the resting brain network templates [36]. The IC selection referred to previous criteria [37]: (1) peak coordinates of ICs located primarily in the gray matter; (2) almost no spatial overlap with blood vessels, ventricles, and suspicious artifacts; (3) dominated by low-frequency signals (<0.1 Hz). Following these steps, 11 ICs were kept for further analysis and arranged into different functional networks, including the default mode network (DMN), sensorimotor network (SMN), dorsal attention network (DAN), left frontoparietal network (LFPN), and right frontoparietal network (RFPN). And then, we performed the following postprocessing steps on the time courses of 11 ICs: (1) detrending linear, quadratic, and cubic trends; (2) removing spikes; (3) low-pass filtering with a cut-off frequency of 0.15 Hz. To estimate functional network connectivity, Pearson's correlations were performed using postprocessed time courses between ICs throughout the whole scanning and then converted to *z* values using Fisher's *z*-transformation to improve the normality [25, 38].

### 2.8. Dynamic Functional Network Connectivity

#### 2.8.1. Sliding Window Analysis

Sliding window analysis, the most common method in dFNC analysis, was used to construct brain functional network in GIFT toolbox. Previous studies had confirmed that the window length of 20-30 TR can better reflect the dynamic characteristics of the brain [39, 40]. Thus, we selected a window length of 20TR, in step of 1TR, computing 149 individual windows. These time windows were convolved with a Gaussian value of *σ* = 3 TRs and computed via the L1-regularized precision matrix. To obtain *z* values and stabilize variance for further analyses, Fisher's *z*-transformation was applied to the functional connectivity matrices.

#### 2.8.2. Clustering Analysis

Based on the windowed functional connectivity matrices of all participants, a *k*-means clustering algorithm was applied to detect reoccurring FC patterns. We used the city-block distance with 500 iterations and 150 replicates to assess the similarity between all time windows. The optimal number of clustering states was estimated by the elbow criterion. The number was determined to be 3, which meant 3 FC states, exhibiting different FC patterns.

#### 2.8.3. Temporal Properties and Connectivity Strength of Dynamics States

Three parameters were measured to assess the temporal properties of FNC: (1) fraction time (the frequency of each state); (2) mean dwell time (the average time a subject spent in any state without converting to another state); (3) number of transitions (switching times between different states). Besides, the connectivity strength of 3 states within the TATG group and the SATG group was also calculated. Analysis of covariance (ANCOVA) was used to compare the mean changes from before treatment to after treatment between groups. Disease duration and lesion volume were taken as covariates. And paired samples *t*-test was adopted to compare the changes before and after treatment in each group. The *P* value for the above analysis was set to 0.05 and corrected for false discovery rate (FDR) correction.

### 2.9. Correlation Analysis

Pearson's correlation was carried out to assess the relationship between clinical characteristics and dFNC parameters at baseline in all subjects, as well as FC values showing significant changes in any state before and after treatment (level of significance: *P* < 0.05).

### 2.10. Validation Analysis

We used different sliding window lengths and number of clusters to verify the stability of our results. The numbers of clusters were set at 4 and 5, and the sliding window lengths were set at 22TRs and 30TRs (detailed information in Supplementary Material 2).

## 3. Results

### 3.1. Demographic Characteristics

A total of 40 patients completed the whole study, including 26 patients in the TATG group and 14 patients in the SATG group. There was no significant difference in age, gender, and duration of disease between the TATG group and the SATG group (*P* > 0.05). The detailed data is presented in Table 1.

### 3.2. Clinical Information

For the clinical data, no statistical differences were observed in NIHSS and FMA scores pretreatment and posttreatment between the two groups (all *P* > 0.05). Intragroup comparisons showed that FMA-lower score, FMA total score, and NIHSS score improved after treatment compared with those before treatment in both the TATG group and the SATG group (all *P* < 0.05). In addition, the FMA-upper score increased after treatment in the TATG group (*P* < 0.05), while this result was not found in the SATG group (*P* > 0.05). The clinical information was provided in Table 2.

### 3.3. Intrinsic Connectivity Networks and Dynamic Functional Connectivity States

**Figure 2:**
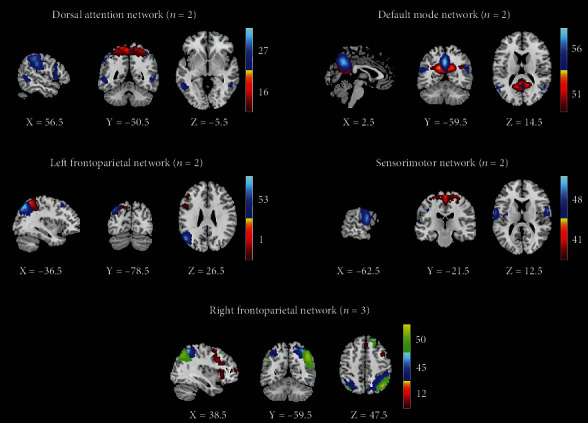
Spatial maps of all 11 independent components. They were assigned to 5 networks: the default mode network, the sensorimotor network, the dorsal attention network, the left frontoparietal network, and the right frontoparietal network. Different color represents the location of independent component of the peak point in each network.

**Figure 3:**
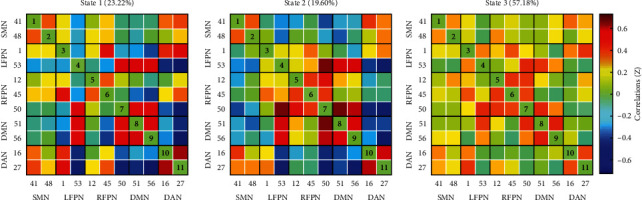
Three different dynamic functional connectivity states. Each matrix represents a stable connectivity state within the data. The percentage of occurrences in each state is also listed. The warm color indicates stronger positive connectivity, and the cold color implies stronger negative connectivity.

We identified five resting-state networks from the 11 ICs, including the default mode network (DMN: ICs 51, 56), the sensorimotor network (SMN: ICs 41, 48), the dorsal attention network (DAN: ICs 16, 27), the left frontoparietal network (LFPN: ICs 1, 53), and the right frontoparietal network (RFPN: ICs 12, 45, 50). Spatial maps of 11 ICs are shown in Figure 2. All FNC matrices were clustered into 3 connectivity states. State 1 presented a relatively complicated connectivity pattern, which was characterized by partly strong positive connections within networks (such as DMN and DAN) and predominantly negative and neutral internetwork connections (between DAN and LFPN, between SMN and other networks, etc.), along with less positive connections (between FPN and DMN). Therefore, we referred to this state as the relative segregated state. The overall frequency of state 1 accounted for 23.22% of all time windows. State 2 seemed to display a more densely intranetwork connection, especially in FPN. We called it the local tightly connected state. The frequency of state 2 was lower than state 1 at 19.60%. State 3 had the longest time window percentage of 57.18%. This state exhibited sparse intra-/internetwork connectivity, which meant no high positive or negative connections within or between networks. And we called this the sparsely connected state. All states are shown in Figure 3.

### 3.4. Temporal Properties

**Figure 4:**
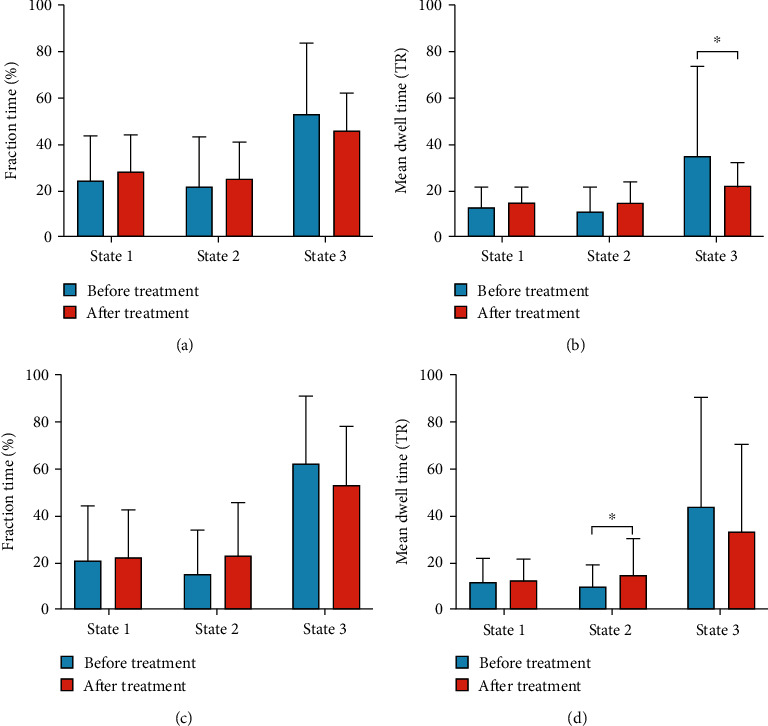
Changes in dynamic properties before and after treatment in the TATG group and the SATG group. (a) Change in fraction time before and after treatment in the TATG group. (b) Change in mean dwell time before and after treatment in the TATG group. (c) Change in fraction time before and after treatment in the SATG group. (d) Change in mean dwell time before and after treatment in the SATG group. TATG: true acupuncture treatment group. SATG: sham acupuncture treatment group. ^∗^ represents *P* < 0.05.

There were no statistical differences observed between the two groups in mean change of fraction time, mean dwell time, and number of transitions. The detailed results are listed in Table 3. The TATG group showed a significantly shorter mean dwell time in state 3 after treatment than that before treatment (*P* < 0.05, FDR correction), while the SATG group revealed a longer mean dwell time in state 2 after treatment (*P* < 0.05, FDR correction). Detailed information are shown in Table 4 and Figure 4.

### 3.5. Strength of Dynamics States

We analyzed the alterations of FC within and between groups for each state. The TATG group showed increased FC between DAN and DMN in state 1 and reduced FC between RFPN and SMN in state 2 and FPN and SMN in state 3 after treatment (all *P* < 0.05). The SATG group had elevated FC in FPN (between LFPN and RFPN, within RFPN) in state 2 after treatment (*P* < 0.05).

**Figure 5:**
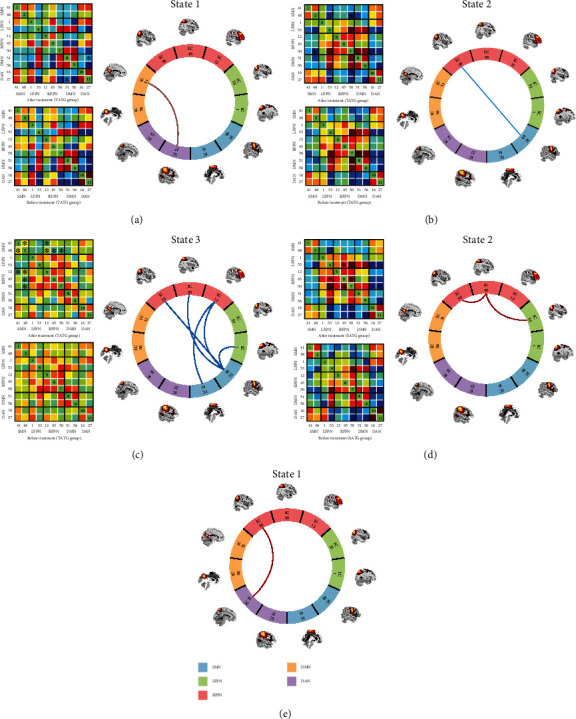
Differences in functional connectivity among distinct states. Specific connectivity matrix (left) and circle plots of significant difference in connectivity states (right) (a–d). The significant difference in specific state is marked by asterisk (^∗^ represents *P* < 0.05). In circle plots, red lines indicate increased functional connectivity (FC) between or within networks. Blue lines indicate decreased FC between or within networks. (a–d) The significant connectivity differences after treatment compared to before treatment in the TATG group or the SATG group. (a) Increased FC between DAN and DMN in state 1 in the TATG group. (b) Reduced FC between RFPN and SMN in state 2 in the TATG group. (c) Reduced FC between FPN and SMN in state 3 in the TATG group. (d) Increased FC in FPN in state 2 in the SATG group. (e) Positive effect between DAN and RFPN in state 1 between the two groups, which means increased FC between DAN and RFPN in state 1 in the TATG group compared to the SATG group. SMN: sensorimotor network. LFPN: left frontoparietal network. RFPN: the right frontoparietal network. DMN: default mode network. DAN: dorsal attention network.

Group difference showed positive effect between DAN and RFPN in state 1 (*P* < 0.05), which means increased FC between DAN and RFPN in state 1 in the TATG group compared to the SATG group. Altered connectivity pairs are shown in Figure 5.

### 3.6. Correlation between Clinical Measures and dFNC Parameters

**Figure 6:**
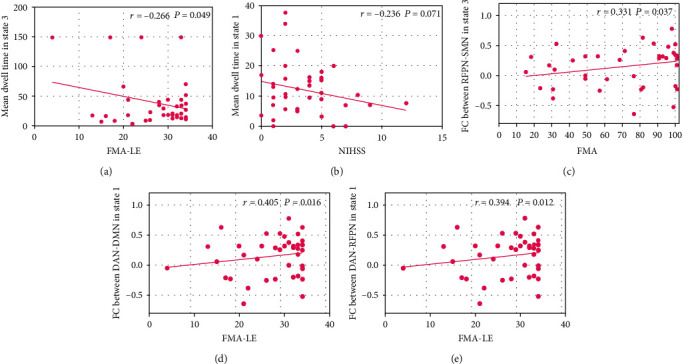
Correlation between clinical measures and dFNC parameter. (a) Negative correlation between mean dwell time and FMA-LE score in state 3. (b) Negative correlation trend between mean dwell time and NIHSS score in state 1. (c) Positive correlation between FMA score and FC in RFPN-SMN in state 3. (d) Positive correlation between FMA-LE score and FC in DAN-DMN in state 1. (e) Positive correlation between FMA-LE score and FC in DAN-RFPN in state 1. FMA: Fugl-Meyer. FMA-LE: FMA for the lower extremity. NIHSS: National Institute of Health Stroke Scale.

Correlation analyses were carried out to detect whether dFNC parameters were related to clinical characteristics. The mean dwell time was negatively correlated with FMA-lower score in state 3 (*r* = −0.266, *P* = 0.049). We speculate that better motor recovery may be associated with short time spent in state 3 to some extent. We also found a negative correlation trend between mean dwell time and NIHSS score in state 1 (*r* = −0.236, *P* = 0.071). In addition, FMA score showed positive correlation with FC in RFPN-SMN in state 3 (*r* = 0.331, *P* = 0.037). FMA-lower score was positively correlated with FC in DAN-DMN and DAN-RFPN in state 1 (*r* = 0.405, *P* = 0.016 and *r* = 0.394, *P* = 0.012) (see in Figure 6).

### 3.7. Validation Analysis

Due to the different settings of dynamic analysis parameters, the results are different to some extent. But most of our main results were well replicated when using different parameters. These observations indicated that acupuncture can indeed improve the abnormal temporal properties and functional network connectivity in stroke patients. One interesting finding was that the number of transitions significantly increased after treatment in the TATG group when the number of clusters *k* = 3 and the length of sliding time windows *W* = 22TRs. Previous studies reported that such variability may lead to greater cognitive and behavioral flexibility [41, 42]. Thus, we speculated that true acupuncture has the potential to improve the brain's flexibility.

## 4. Discussion

### 4.1. Acupuncture Improves Motor Function in Stroke Patients with Hemiplegia

From Traditional Chinese Medicine, *Shou Zu Shi Er Zhen* has the effect of harmonizing qi and blood, balancing yin and yang, harmonizing the spleen and stomach, and regulating body constituent and spirit [30, 43]. Current evidence suggested that conventional treatment combined with *Shou Zu Shi Er Zhen* therapy is more effective in improving FMA score and lowering NIHSS score compared to conventional treatment in patients with poststroke hemiplegia, exerting the effectiveness of acupuncture [13–15]. In this study, we found significant improvements in the FMA score (FMA-upper, FMA-lower, and FMA total) and NIHSS score in the TATG group after treatment, which is in accordance with previous studies.

### 4.2. Acupuncture Alters Brain's Dynamic Functional Network Connectivity

After treatment, the TATG group showed a reduced mean dwell time in state 3, as manifested sparse connections within and between brain functional networks. Previous studies demonstrated that such weakly connected state resembled stationary functional connectivity, representing the average of a great number of additional states that were infrequent to be separated or insufficiently distinct [25]. Furthermore, recent studies suggested that a significantly longer mean dwell time of this weakly connected state was observed in stroke patients compared to healthy individuals and may prevent the recovery of neurological function [29, 44–46]. With disease remission, the mean dwell time in weakly state decreases and tends to normal time [44]. Our correlation analysis revealed that the motor dysfunction improved with a reduction of mean dwell time in state 3. The stroke patients who received *Shou Zu Shi Er Zhen* treatment displayed a significant decrease in mean dwell time in state 3, indicating the positive effects of acupuncture in ameliorating the abnormal temporal properties. One study considered that a weakly connected pattern was more likely to be altered and converted to another connection pattern or to construct a new function matrix [29]. Hence, we speculate that acupuncture has the potential to enhance such alteration and promote abnormal whole-brain functional connectivity toward normal patterns.

In addition, we found attenuated connectivity between SMN and FPN in state 3 in the TATG group after treatment. SMN is involved in perceptual and motor function, and FPN participates in many cognitive tasks consisting of attention, working memory, and motor control [22, 47]. Several studies report that increased FC between SMN and FPN at earlier time after stroke contributed to complete motor planning and execution by integrating information related to the motor-related goal and spatial information, reflecting a compensation strategy for the damaged motor system [22, 47, 48]. As stroke patients recover, the higher FC between brain networks will be decreased. However, prolonged compensatory brain activity is believed to a maladaptive plasticity, which leads to poorer motor performance [49, 50]. Therefore, acupuncture may facilitate aberrant functional network normalization during stroke rehabilitation.

Besides, we found both fraction time and mean dwell time in state 1 that had an increasing tendency after *Shou Zu Shi Er Zhen* acupuncture treatment. State 1 was characterized by partially high intranetwork connectivity, as well as low internetwork connectivity with few positive connections. We referred to this state as the relative segregated state, which can be explained by the concept of functional segregation [51, 52]. Functional segregation reflects better cognitive abilities and information processing for specific tasks [53]. Recent evidence shows that the mean dwell time of such state significantly decreased in stroke patients, suggesting reduced functional segregation to be a sign of impaired function [45, 54]. In our study, there was a negative trend between NIHSS score and the mean dwell time in state 1, meaning reduced mean dwell time in segregated state in parallel to worse neurological deficits. Nevertheless, this interpretation should be viewed with caution due to limited statistical power. Notably, the connectivity between DAN and DMN in state 1 was positively correlated with FMA score and was higher in the TATG group after treatment than before. DAN is related to motor attention task, especially in spatial attention and pointing movements [55]. DMN is associated with self-referential and spontaneous cognition, and this network is anticorrelated with DAN in the resting state [56]. Neuroimaging evidence revealed that the enhanced negative connectivity may compensate for decreased cognitive function in Parkinson's disease [57]. Prior studies suggested that acupuncture therapy could help patients improve their cognitive function [58, 59]. Our result showed an increased FC between DAN and DMN after true acupuncture treatment; that is, the negative FC between the two networks decreased. Thus, we presume that acupuncture promotes higher-order cognitive information integration without the need for functional compensation induced by increased negative FC between DAN and DMN. And the result also revealed that decreased FC between DAN and DMN was positively related to good motor recovery, supporting our speculation to some extent.

In terms of sham acupuncture treatment, we found increased mean dwell time, as well as elevated FC within FPN in state 2 after treatment. FPN is mainly involved in the cognitive control process such as attention and memory [60]. Fu et al. approved that FPN was the causal hub and inputted most information from other brain networks in the resting state for stroke patients [22]. Interestingly, after needling stimulation, they found that LFPN converted into the network outputting most information to other brain networks, reflecting that acupuncture may help transmit information by thinking and decision-making. Considering our results, we speculated that sham acupuncture also had a certain effect on brain functional activity, which may help to strengthen communication within FPN to support cognitive process and motor preparation.

### 4.3. Differences in Functional Connectivity between True and Sham Acupuncture

Acupuncture points are distributed on the meridians, with specific names, definite locations and clear therapeutic ranges. Acupoint specificity is considered the core scientific issue in the practice of acupuncture at the Society for Acupuncture Research International Symposium held in 2007 [31]. Several studies have demonstrated that acupoint stimulation in stroke patients induces significant activation of more brain areas and stronger effective connections between functional networks in comparison to sham acupuncture [61]. We found that the FC value increased between DAN and RFPN in state 1 in the TATG group compared to the SATG group. And further analysis showed that stronger FC between DAN and RFPN was associated with better motor performance. Accordingly, we supposed that true acupuncture may enhance the integration of movement-related and cognitive formation. In terms of temporal properties, there was a trend toward a greater decline in mean dwell time in the weakly connected state (state 3), as well as a larger increase in mean dwell time in the relative segregated state (state 1) in patients treated with true acupuncture compared to sham acupuncture, and these alterations were closely related with motor recovery. Interestingly, the mean dwell time in state 2 increased significantly after sham acupuncture, with elevated FC within FPN in state 2. However, no clinical relevance was observed for these indicators in state 2. Such changes suggest that sham acupoint stimulation also has some impact on brain function but may not be sufficient to achieve therapeutic effects.

### 4.4. Limitations

This work has several limitations. In this study, we failed to confirm the advantages of true acupuncture from the clinical perspective. Some evidence suggests that sham acupuncture also creates some effects, which may underestimate the true acupuncture treatment effect [11]. And although the sample size met the requirement of a minimum sample size for statistical power in neuroimaging research [62, 63], it was still relatively small for a clinical study. Our neuroimaging results also need to be validated via independent datasets with larger sample sizes. Besides, due to the limitation of the objective conditions, we only performed two weeks of acupuncture intervention for stroke patients, which is not the optimal duration of treatment for rehabilitation. Further studies should provide long-course acupuncture treatment to explore the mechanism underlying how acupuncture promotes brain neuroplasticity. In addition, there is evidence suggesting that stroke severity is related to temporal properties and dynamic connectivity of the brain. In the present study, most patients were mild to moderate, and our conclusions may not be adapted to patients with severe motor dysfunction.

## 5. Conclusion

This is the first study to investigate the alteration of dynamic brain function induced by acupuncture treatment in stroke patients with motor dysfunction based on dFNC analysis. We identified the relationship between dynamic properties and motor function in poststroke patients. Our findings demonstrated that acupuncture has the potential to modulate abnormal temporal properties and promote the balance of separation and integration of brain function. True acupoint stimulation may have a more positive effect on regulating the brain's dynamic function. Our findings provide additional evidence for exploring the association between acupuncture therapy and neuroplasticity.

## Figures and Tables

**Table 1:** Demographic features of stroke patients.

	The TATG group (*n* = 26)	The SATG group (*n* = 14)	*P* value
Sex (male/female)	18/8	10/4	0.885
Age (years)	59.38 ± 11.61	59.14 ± 8.77	0.946
Disease duration (days)	15.89 ± 20.70	20.07 ± 15.34	0.831
Lesion side (left/right)	12/14	6/8	0.842
Lesion volume (ml)	2.96 ± 2.85	4.75 ± 3.97	0.127

Values were expressed as mean ± standard deviation. TATG: true acupuncture treatment group; SATG: sham acupuncture treatment group. The chi-square test was used to compare the distribution of sex and lesion side between the two groups.

**Table 2:** Clinical assessment before and after treatment in the two groups.

Clinical scales	The TATG group			The SATG group		
	Before treatment	After treatment	*P* value	Before treatment	After treatment	*P* value
FMA-UE	41.23 ± 23.87	46.42 ± 22.57	<0.0001^∗^	45.50 ± 24.49	49.42 ± 23.39	0.058
FMA-LE	27.50 ± 7.58	30.19 ± 5.06	<0.0001^∗^	27.64 ± 7.08	30.64 ± 4.49	0.013^∗^
FMA	68.73 ± 37.38	76.61 ± 25.65	<0.0001^∗^	73.14 ± 30.71	80.07 ± 27.56	0.016^∗^
NIHSS	3.46 ± 2.42	2.46 ± 2.24	<0.0001^∗^	3.64 ± 3.24	2.50 ± 3.05	0.002^∗^

Values were expressed as mean ± standard deviation. TATG: true acupuncture treatment group; SATG: sham acupuncture treatment group. FMA: Fugl-Meyer. FMA-UE: FMA for the upper extremity. FMA-LE: FMA for the lower extremity. NIHSS: National Institute of Health Stroke Scale. The *P* value represents the difference before and after treatment in the TATG group or the SATG group. ^∗^ represents *P* < 0.05.

**Table 3:** Comparison in temporal properties between the two groups.

	Fraction time (%)			Mean dwell time (TR)			Number of transitions
	State 1	State 2	State 3	State 1	State 2	State 3	
Mean difference (TATG-SATG)	4.900	-4.200	-0.600	1.919	-2.966	-6.886	1.503
95% confidence interval (lower bound, upper bound)	-10.50020.200	-17.500 9.000	-15.300, 14.000	-6.391, 10.230	-10.107, 4.175	-31.153, 17.380	-1.335, 4340
*F*	0.417	0.428	0.008	0.221	0.716	0.334	1.164
*P*	0.523	0.518	0.931	0.641	0.404	0.567	0.289

TATG: true acupuncture treatment group; SATG: sham acupuncture treatment group.

**Table 4:** Comparison in temporal properties within the two groups.

		Fraction time (%)			Mean dwell time (TR)			Number of transitions
		State 1	State 2	State 3	State 1	State 2	State 3	
The TATG group	Before treatment	24.08 ± 19.70	21.73 ± 21.51	54.18 ± 29.54	12.38 ± 8.54	11.06 ± 9.55	35.08 ± 38.59	6.92 ± 3.42
	After treatment	28.55 ± 15.55	24.96 ± 15.71	46.49 ± 15.94	14.61 ± 6.88	14.25 ± 9.03	21.35 ± 10.97	8.11 ± 2.59
	*t*	-1.133	-0.805	1.716	-1.068	-1.456	2.096	-1.563
	*P*	0.268	0.428	0.098	0.296	0.158	0.046^∗^	0.131

The SATG group	Before treatment	21.62 ± 21.92	15.63 ± 18.23	62.75 ± 28.28	11.39 ± 10.63	8.74 ± 10.70	44.37 ± 46.14	6.5 ± 3.83
	After treatment	22.82 ± 19.67	23.30 ± 23.09	53.88 ± 23.96	12.42 ± 8.53	15.14 ± 15.60	33.50 ± 36.39	6.5 ± 3.27
	*t*	-0.189	-1.588	1.622	-0.296	-2.313	1.113	0
	*P*	0.853	0.136	0.129	0.772	0.038^∗^	0.286	1

Values were expressed as mean ± standard deviation. TATG: true acupuncture treatment group; SATG: sham acupuncture treatment group. The *P* value represents the difference before and after treatment in the TATG group or the SATG group. ^∗^ represents *P* < 0.05.

## Data Availability

The data used to support the findings of this study will be available upon reasonable request, which can be directed to the corresponding author.

## Supplementary Materials

Supplementary Materials The supplementary materials provide more detailed information. The probability map and detailed information of the lesion location in each patient are shown in Supplementary Material 1. The location of all acupoints is also shown in Supplementary Material 1. The results of validation analysis are shown in Supplementary Material 2.
