# Supplementary material for: Acupuncture Alters Brain's Dynamic Functional Network Connectivity in Stroke Patients with Motor Dysfunction: A Randomised Controlled Neuroimaging Trial
Source: Neural Plast. 2023 Jun 20;2023:8510213. doi: 10.1155/2023/8510213 (PMC10299883; doi:10.1155/2023/8510213)
Supplement: Supplementary Materials — The supplementary materials provide more detailed information. The probability map and detailed information of the lesion location in each patient are shown in Supplementary Material 1. The location of all acupoints is also shown in Supplementary Material 1. The results of validation analysis are shown in Supplementary Material 2. [file 8510213.f1.zip › Supplementary material 1.doc]

The detail information of location of the lesions in each patient

| No. | sex | age | hemisphere | location |
| --- | --- | --- | --- | --- |
| 01 | male | 49 | right | posterior limb of internal capsule |
| 02 | male | 60 | right | thalamus |
| 03 | male | 55 | right | corona radiata |
| 04 | male | 63 | right | posterior limb of internal capsule |
| 05 | male | 52 | left | basal ganglia |
| 06 | male | 66 | left | posterior limb of internal capsule |
| 07 | female | 53 | right | periventricular |
| 08 | female | 69 | left | periventricular and posterior limb of internal capsule |
| 09 | male | 68 | right | centrum semiovale and thalamus |
| 10 | male | 38 | right | thalamus |
| 11 | male | 51 | left | periventricular |
| 12 | male | 35 | left | basal ganglia |
| 13 | male | 38 | right | basal ganglia |
| 14 | female | 71 | right | internal capsule |
| 15 | female | 73 | right | basal ganglia and periventricular |
| 16 | male | 60 | left | basal ganglia |
| 17 | male | 77 | right | periventricular and centrum semiovale |
| 18 | male | 62 | left | periventricular |
| 19 | female | 62 | left | periventricular and basal ganglia |
| 20 | male | 68 | left | basal ganglia and periventricular |
| 21 | male | 65 | right | basal ganglia |
| 22 | female | 43 | left | periventricular |
| 34 | male | 75 | right | basal ganglia and corona radiata |
| 35 | female | 68 | left | basal ganglia and corona radiata |
| 36 | male | 61 | left | basal ganglia and corona radiata |
| 39 | female | 62 | right | periventricular |
| 23 | male | 73 | left | periventricular |
| 24 | male | 59 | right | basal ganglia |
| 25 | male | 43 | left | periventricular and basal ganglia |
| 26 | male | 65 | right | periventricular |
| 27 | male | 57 | right | periventricular |
| 28 | male | 47 | right | periventricular |
| 29 | male | 53 | right | periventricular |
| 30 | female | 57 | right | periventricular |
| 31 | female | 59 | left | basal ganglia and periventricular |
| 32 | male | 52 | left | periventricular and basal ganglia |
| 33 | female | 64 | right | thalamus |
| 37 | male | 72 | left | basal ganglia and periventricular |
| 38 | male | 59 | right | basal ganglia and periventricular |
| 40 | female | 68 | left | basal ganglia and corona radiata |

**The location of all acupoints.** Red dots indicate true points in the TATG group and black dots indicates sham points in the SATG group.

**The probability map of the lesion location.** CL: contralesional; IL: ipsilesional. The color bar represents the number of subjects in which voxels lay within stroke lesion.
