# Supplementary material for: Acupuncture Alters Brain's Dynamic Functional Network Connectivity in Stroke Patients with Motor Dysfunction: A Randomised Controlled Neuroimaging Trial
Source: Neural Plast. 2023 Jun 20;2023:8510213. doi: 10.1155/2023/8510213 (PMC10299883; doi:10.1155/2023/8510213)
Supplement: Supplementary Materials — The supplementary materials provide more detailed information. The probability map and detailed information of the lesion location in each patient are shown in Supplementary Material 1. The location of all acupoints is also shown in Supplementary Material 1. The results of validation analysis are shown in Supplementary Material 2. [file 8510213.f1.zip › Supplementary material 2.doc]

We use different sliding window lengths and number of clusters to verify the stability of the main results. The Pearson correlation analysis was conducted to calculate the similarity between the two states obtained from different parameters. And then we choose the two states with the highest correlation coefficients as the corresponding states. Following this, we calculated the temporal properties, connectivity strength, group difference of corresponding states, and assess the relationship between clinical characteristics and dFNC parameters. Then we compared those results with the main results. The parameters and corresponding results of validation analysis are as follows: (1) The number of clusters k=4, and the length of sliding time windows W=20TRs;

(2) The number of clusters k=5, and the length of sliding time windows W=20TRs; (3) The number of clusters k=3, and the length of sliding time windows W=22TRs; (4) The number of clusters k=3, and the length of sliding time windows W=30TRs. We found that the main results had satisfactory repeatability when using different parameters.

**The results are as follows:**

(1) **When the number of clusters k=4, and the length of sliding time windows W=20TRs:**

The state 1 corresponds to state 1 of our study (r=0.998, p=0.000), state 3 corresponds to state 2 of our study (r=0.999, p=0.000) and state 4 corresponds to state 3 of our study (r=0.973, p=0.000). The TATG group showed a significantly shorter fraction time and mean dwell time in state 4 after treatment than that before treatment (*P* < 0.05, FDR correction), while the SATG group revealed a longer mean dwell time in state 3 after treatment (*P* < 0.05, FDR correction). No statistical differences were found between the two groups. The TATG group showed increased FC between DAN and DMN, between DMN and SMN in state 1, increased FC between DMN and SMN, RFPN and SMN in state 3, increased FC within RFPN, reduced FC SMN and FPN in state 4 after treatment（all *P* < 0.05). The SATG group had elevated FC in FPN (between LFPN and RFPN) in state 2 after treatment (*P* < 0.05). Group difference was observed between DAN and RFPN in state 1 (*P* < 0.05), which means increased FC between DAN and RFPN in state 1 in the TATG group compared to the SATG group. We also found a negative correlation trend between fraction time and FMA lower score in state 4 ( *r* = -0.221, *P* =0.085).

**(2) The number of clusters k=5, and the length of sliding time windows W=20TRs;**

The state 1 corresponds to state 3 of our study (r=0.958, p=0.000), state 4 corresponds to state 1 of our study (r=0.995, p=0.000) and state 5 corresponds to state 2 of our study (r=0.928, p=0.000). The TATG group showed a significantly shorter fraction time in state 1 and longer mean dwell time in state 4 after treatment than that before treatment (*P* < 0.05, FDR correction), while the SATG group revealed a longer mean dwell time in state 5 after treatment (*P* < 0.05, FDR correction). No statistical differences were found between the two groups. The TATG group showed increased FC (within RFPN) and reduced FC (SMN -FPN) in state 1,increased FC (DMN-DAN) in state 4, reduced FC (RFPN-SMN) in state 5 after treatment. The SATG group had elevated FC (between LFPN and RFPN) and reduced FC (LFPN-DAN) in state 2 after treatment (all *P* < 0.05). Group difference was observed between DAN and RFPN in state 1 (*P* < 0.05), which means increased FC between DAN and RFPN in state 1 in the TATG group compared to the SATG group. We found a negative correlation trend between mean dwell time and FMA lower score in state 1 ( *r* = -0.21, *P* =0.031).

**(3) The number of clusters k=3, and the length of sliding time windows W=22TRs;**

The state 1 corresponds to state 3 of our study (r=0.999, p=0.000), state 2 corresponds to state 2 of our study (r=0.999, p=0.000) and state 3 corresponds to state 1 of our study (r=0.999, p=0.000). The TATG group showed a significantly shorter fraction time in state 1 and increased number of transitions after treatment than that before treatment (*P* < 0.05, FDR correction). No statistical differences were found within SATG group and between the two groups. The TATG group showed reduced FC (DMN-RFPN, SMN-FPN) in state 1,increased FC (DMN-DAN) in state 2, increased FC (DMN-DAN) and reduced FC (DAN-LFPN) in state 3 after treatment. The SATG group had elevated FC (LFPN-DAN) in state 2 after treatment (all *P* < 0.05). Group difference was observed between DAN and RFPN in state 1 (*P* < 0.05), which means increased FC between DAN and RFPN in state 1 in the TATG group compared to the SATG group. We found a negative correlation trend between mean dwell time and FMA lower score in state 1 ( *r* = -0.266, *P* =0.048).

**(4) The number of clusters k=3, and the length of sliding time windows W=30TRs.**

The state 1 corresponds to state 3 of our study (r=0.990, p=0.000), state 2 corresponds to state 1 of our study (r=0.985, p=0.000) and state 3 corresponds to state 2 of our study (r=0.967, p=0.000). The TATG group showed a significantly longer mean dwell time in state 2 after treatment than that before treatment (*P* < 0.05, FDR correction). No statistical differences were found within SATG group and between the two groups. The TATG group showed reduced FC (SMN-FPN) in state 1, increased FC (DMN-DAN) in state 2 after treatment. The SATG group had elevated FC (RFPN-DMN,RFPN -LFPN,within LFPN) and reduced FC (SMN-RFPN, DAN-LFPN) in state 2 after treatment (all *P* < 0.05). Group difference was observed between DAN and RFPN in state 1 (*P* < 0.05), which means increased FC between DAN and RFPN in state 1 in the TATG group compared to the SATG group. No correlation was found between tempral properties and FMA score in all states.

**Table S.1-4 shows** the correlation coefficient (r) and *p*-values between validation analyses and those in our analysis.

**Figure S.1-4** displays the centroid of each functional network connectivity state.

**Table S.5-8** and **Figure S.5-8** shows the temporal properties and connectivity strength of dFNC within each group using different sliding window lengths and number of clusters.

**Table S.9-12** shows comparison in temporal properties between the two groups.

**Figure S.9-12** shows Group difference of FC in brain networks between the TATG group and the SATG group

**Table S.1** The correlation coefficient between States when the number of clusters is 3 and 4.

| k=3  W=20TR | k=4 W=20TR | | | |
| --- | --- | --- | --- | --- |
|  | state1 | state2 | state3 | state4 |
| state1 | **r=0.998***  (P=0.000) | r=0.738  (P=0.000) | r=0.681  (P=0.000) | r=0.729  (P=0.000) |
| state2 | r=0.680  (P=0.000) | r=0.783  (P=0.000) | **r=0.999***  (P=0.000) | r=0.734  (P=0.000) |
| state3 | r=0.693  (P=0.000) | r=0.930  (P=0.000) | r=0.733  (P=0.000) | **r=0.973***  (P=0.000) |

**Table** **S.2** The correlation coefficient between States when the number of clusters is 3 and 5.

| k=3  W=20TR | k=5 W=20TR | | | | |
| --- | --- | --- | --- | --- | --- |
|  | state1 | state2 | state3 | state4 | state5 |
| state1 | r=0.740  (P=0.000) | r=0.709  (P=0.000) | r=0.740  (P=0.000) | **r=0.995***  (P=0.000) | 0.628  (P=0.000) |
| state2 | r=0.660  (P=0.000) | r=0.892  (P=0.000) | r=0.773  (P=0.000) | r=0.658  (P=0.000) | **r=0.928***  (P=0.000) |
| state3 | **r=0.958***  (P=0.000) | r=0.671  (P=0.000) | r=0.931  (P=0.000) | r=0.681  (P=0.000) | r=0.758  (P=0.000) |

**Table S.3** The correlation coefficient between States when the length of sliding time windows is 20 and 22 TRs.

| k=3  W=20TR | k=3 W=22TR | | |
| --- | --- | --- | --- |
|  | state1 | state2 | state3 |
| state1 | r=0.704  (P=0.000) | r=0.685  (P=0.000) | **r=0.999***  (P=0.000) |
| state2 | r=0.763  (P=0.000) | **r=0.999***  (P=0.000) | r=0.687  (P=0.000) |
| state3 | **r=0.999***  (P=0.000) | r=0.746  (P=0.000) | r=0.721  (P=0.000) |

**Table S.4** The correlation coefficient between States when the length of sliding time windows is 20 and 30 TRs.

| k=3  W=20TR | k=3 W=30TR | | |
| --- | --- | --- | --- |
|  | state1 | state2 | state3 |
| state1 | r=0.702  (P=0.000) | **r=0.985***  (P=0.000) | r=0.784  (P=0.000) |
| state2 | r=0.807  (P=0.000) | r=0.679  (P=0.000) | **r=0.967***  (P=0.000) |
| state3 | **r=0.990***  (P=0.000) | r=0.744  (P=0.000) | r=0.744  (P=0.000) |

**Table S.5** The temporal properties within the two groups when the number of clusters k=4, and the length of sliding time windows W=20TRs.

|  |  | Fraction time (%) | | | | Mean dwell time (TR) | | | | Number of transitions |
| --- | --- | --- | --- | --- | --- | --- | --- | --- | --- | --- |
|  |  | state1 | state2 | state3 | state4 | state1 | state2 | state3 | state4 |  |
| The TATG group | Before treatment | 20.52±18.40 | 31.85±21.50 | 20.42±21.86 | 27.21±23.08 | 11.66±8.39 | 14.64±10.06 | 11.35±10.17 | 14.27±9.10 | 8.65±2.38 |
|  | After treatment | 24.21±15.60 | 36.55±18.31 | 22.48±16.89 | 16.75±15.95 | 14.42±6.76 | 18.35±11.53 | 14.73±10.56 | 11.80±9.94 | 8.53±3.04 |
|  | t | -1.003 | -1.12 | -0.481 | 2..461 | -1.699 | -1.311 | -1.543 | 0.984 | 0.144 |
|  | P | 0.325 | 0.273 | 0.635 | **0.021*** | 0.102 | 0.202 | 0.136 | **0.035*** | 0.886 |
| The SATG group | Before treatment | 17.16±20.61 | 45.54±22.47 | 12.61±18.15 | 24.69±20.35 | 8.57±7.51 | 20.87±10.48 | 8.54±11.81 | 12.86±9.48 | 8.50±3.25 |
|  | After treatment | 19.22±17.50 | 40.27±18.44 | 21.24±20.98 | 19.27±18.60 | 12.81±12.18 | 18.51±15.29 | 15.19±14.81 | 15.11±11.29 | 8.00±3.53 |
|  | t | -0.345 | 0.813 | -2.03 | 0.845 | -1.148 | 0.454 | -2.951 | -0.611 | 0.411 |
|  | P | 0.736 | 0.431 | 0.063 | 0.413 | 0.271 | 0.657 | **0.011*** | 0.552 | 0.688 |

Values were expressed as mean ± standard deviation. TATG: true acupuncture treatment group, SATG: sham acupuncture treatment group. P-value represents the difference before and after treatment in the TATG group or the SATG group. *Represents P < 0.05.

**Table S.6** The temporal properties within the two groups when the number of clusters k=5, and the length of sliding time windows W=20TRs.

|  |  | Fraction time (%) | | | | | Mean dwell time (TR) | | | | | Number of transitions |
| --- | --- | --- | --- | --- | --- | --- | --- | --- | --- | --- | --- | --- |
|  |  | state1 | state2 | state3 | state4 | state5 | state1 | state2 | state3 | state4 | state5 |  |
| The TATG group | Before treatment | 25.30±21.91 | 18.87±20.87 | 28.27±19.50 | 15.23±16.63 | 12.34±14.68 | 13.25±8.78 | 9.44±8.79 | 12.75±8.87 | 7.82±7.44 | 9.39±8.66 | 10.00±2.59 |
|  | After treatment | 15.51±15.44 | 16.37±20.25 | 30.23±17.70 | 20.96±15.11 | 16.93±16.78 | 11.11±9.58 | 11.68±14.43 | 14.77±8.07 | 13.13±7.77 | 12.20±13.86 | 9.46±2.88 |
|  | t | 2.432 | 0.595 | -0.496 | -1.544 | -1.704 | 0.944 | -0.904 | -0.845 | -3.564 | -0.978 | 0.748 |
|  | P | **0.023*** | 0.557 | 0.624 | 0.135 | 0.101 | 0.354 | 0.375 | 0.406 | **0.002*** | 0.337 | 0.461 |
| The SATG group | Before treatment | 23.39±19.41 | 15.29±19.72 | 40.56±20.88 | 14.19±19.97 | 6.57±9.26 | 12.98±8.98 | 8.76±8.92 | 16.33±7.90 | 7.14±7.71 | 4.07±4.98 | 9.85±2.50 |
|  | After treatment | 17.35±17.80 | 15.29±16.39 | 36.10±21.72 | 16.87±16.58 | 14.38±16.72 | 14.51±11.06 | 11.56±10.33 | 17.49±14.83 | 11.67±11.55 | 10.15±9.49 | 8.21±2.93 |
|  | t | 1.012 | 0 | 0.761 | -0.466 | -1.619 | -0.422 | -0.991 | -0.275 | -1.347 | -2.331 | 1.562 |
|  | P | 0.33 | 1 | 0.46 | 0.649 | 0.13 | 0.68 | 0.34 | 0.788 | 0.201 | **0.036*** | 0.142 |

Values were expressed as mean ± standard deviation. TATG: true acupuncture treatment group, SATG: sham acupuncture treatment group. P-value represents the difference before and after treatment in the TATG group or the SATG group. *Represents P < 0.05.

**Table S.7** The temporal properties within the two groups when the number of clusters k=3, and the length of sliding time windows W=22TRs.

|  |  | Fraction time (%) | | | Mean dwell time (TR) | | | Number of transitions |
| --- | --- | --- | --- | --- | --- | --- | --- | --- |
|  |  | state1 | state2 | state3 | state1 | state2 | state3 |  |
| The TATG group | Before treatment | 53.56±30.59 | 21.77±23.24 | 24.67±21.18 | 34.79±37.49 | 13.54±13.44 | 14.26±9.52 | 5.84±2.82 |
|  | After treatment | 45.92±16.20 | 24.80±16.87 | 29.82±16.30 | 21.89±10.99 | 13.60±8.59 | 15.38±7.93 | 7.84±2.47 |
|  | t | 1.606 | -0.703 | -1.091 | 2.021 | -0.02 | -0.49 | -3.103 |
|  | P | 0.121 | 0.488 | 0.285 | **0.048*** | 0.984 | 0.629 | **0.005*** |
| The SATG group | Before treatment | 63.36±29.32 | 14.29±19.42 | 22.35±23.94 | 45.93±45.22 | 9.13±12.03 | 12.62±15.21 | 5.78±3.37 |
|  | After treatment | 53.30±25.17 | 22.98±23.10 | 23.71±21.10 | 41.07±46.73 | 15.33±15.88 | 13.03±9.95 | 5.57±3.52 |
|  | t | 1.697 | -1.825 | -0.205 | 0.404 | -1.984 | -0.09 | -0.197 |
|  | P | 0.113 | 0.091 | 0.841 | 0.693 | 0.069 | 0.929 | 0.847 |

Values were expressed as mean ± standard deviation. TATG: true acupuncture treatment group, SATG: sham acupuncture treatment group. P-value represents the difference before and after treatment in the TATG group or the SATG group. *Represents P < 0.05.

**Table S.8** The temporal properties within the two groups when the number of clusters k=3, and the length of sliding time windows W=30TRs.

|  |  | Fraction time (%) | | | Mean dwell time (TR) | | | Number of transitions |
| --- | --- | --- | --- | --- | --- | --- | --- | --- |
|  |  | state1 | state2 | state3 | state1 | state2 | state3 |  |
| The TATG group | Before treatment | 51.83±34.96 | 16.69±23.66 | 31.49±30.37 | 41.24±42.12 | 9.82±12.17 | 22.63±27.45 | 4.15±2.67 |
|  | After treatment | 46.21±19.89 | 25.48±20.37 | 28.31±21.01 | 34.84±24.41 | 16.19±10.82 | 18.79±13.57 | 4.92±1.87 |
|  | t | 0.955 | -1.618 | 0.564 | 0.867 | -2.175 | 0.685 | -1.206 |
|  | P | 0.349 | 0.118 | 0.578 | 0.394 | **0.039*** | 0.500 | 0.239 |
| The SATG group | Before treatment | 60.64±32.58 | 19.22±23.88 | 20.14±24.93 | 46.63±43.53 | 11.77±15.67 | 18.30±29.17 | 4.21±3.09 |
|  | After treatment | 53.03±26.30 | 22.25±19.40 | 24.72±22.90 | 41.55±42.88 | 12.27±10.52 | 16.45±15.28 | 4.85±2.79 |
|  | t | 1.073 | -0.512 | -1.168 | 0.385 | -0.122 | 0.365 | -0.602 |
|  | P | 0.303 | 0.617 | 0.264 | 0.706 | 0.905 | 0.721 | 0.557 |

Values were expressed as mean ± standard deviation. TATG: true acupuncture treatment group, SATG: sham acupuncture treatment group. P-value represents the difference before and after treatment in the TATG group or the SATG group. *Represents P < 0.05.

**Table S.9** Comparison in temporal properties between the two groups when the number of clusters k=4, and the length of sliding time windows W=20TRs.

|  | Fraction time (%) | | | | Mean dwell time (TR) | | | | Number of transitions |
| --- | --- | --- | --- | --- | --- | --- | --- | --- | --- |
|  | state1 | state2 | state3 | state4 | state1 | state2 | state3 | state4 |  |
| Mean difference （TATG - SATG） | 3.866 | -3.113 | -3.529 | 0.161 | 0.993 | -3.507 | -2.798 | -.517 | 0.535 |
| 95% Confidence interval [Lower Bound, Upper Bound] | -6.644, 14.375 | -13.985, 7.760 | -14.611, 7.553 | -12.243, 12.565 | -5.166, 7.153 | -10.693, 3.679 | -9.205, 3.608 | -9.931, 8.897 | -1.678, 2.749 |
| *F* | 0.557 | 0.337 | 0.417 | 0.001 | 0.107 | 0.979 | 0.785 | 0.012 | 0.240 |
| *P* | 0.461 | 0.565 | 0.523 | 0.979 | 0.745 | 0.329 | 0.382 | 0.912 | 0.627 |

TATG: true acupuncture treatment group, SATG: sham acupuncture treatment group.

**Table S.10** Comparison in temporal properties between the two groups when the number of clusters k=5, and the length of sliding time windows W=20TRs.

|  | Fraction time (%) | | | | | | Mean dwell time (TR) | | | | | Number of transitions |
| --- | --- | --- | --- | --- | --- | --- | --- | --- | --- | --- | --- | --- |
|  | state1 | state2 | state3 | state4 | state5 | state1 | | state2 | state3 | state4 | state5 |  |
| Mean difference （TATG - SATG） | -0.329 | -0.768 | -2.388 | 3.826 | 0.206 | -2.905 | | -0.283 | -3.447 | 1.206 | 0.042 | 1.245 |
| 95% Confidence interval [Lower Bound, Upper Bound] | -12.898, 12.241 | -11.798, 10.263 | -12.747, 7.971 | -6.515, 14.167 | -10.325,10.737 | -9.798, 5.607 | | -8.287,7.721 | -10.334, 3.439 | -4.733, 7.145 | -9.838, 9.922 | -0.755, 3.245 |
| *F* | 0.003 | 0.020 | 0.219 | 0.563 | 0.002 | 0.304 | | 0.005 | 1.031 | 0.170 | 0.000 | 1.595 |
| *P* | 0.958 | 0.889 | 0.643 | 0.458 | 0.969 | 0.585 | | 0.943 | 0.317 | 0.683 | 0.993 | 0.215 |

TATG: true acupuncture treatment group, SATG: sham acupuncture treatment group.

**Table S.11** Comparison in temporal properties between the two groups when the number of clusters k=3, and the length of sliding time windows W=22TRs.

|  | Fraction time (%) | | | Mean dwell time (TR) | | | Number of transitions |
| --- | --- | --- | --- | --- | --- | --- | --- |
|  | state1 | state2 | state3 | state1 | state2 | state3 |  |
| Mean difference （TATG - SATG） | -2.522 | -2.801 | 4.975 | -14.636 | -4.453 | 2.219 | 2.258 |
| 95% Confidence interval [Lower Bound, Upper Bound] | -13.071,8.027 | -14.051, 8.450 | -6.622, 16.572 | -31.722, 2.451 | -11.292, 2.387 | -3.752, 8.190 | 0.3455, 4.171 |
| *F* | 0.235 | 0.255 | 0.757 | 3.018 | 1.743 | 0.568 | 5.729 |
| *P* | 0.631 | 0.617 | 0.390 | 0.091 | 0.195 | 0.456 | **0.022** |

TATG: true acupuncture treatment group, SATG: sham acupuncture treatment group.

**Table S.12** Comparison in temporal properties between the two groups when the number of clusters k=3, and the length of sliding time windows W=30TRs.

|  | Fraction time (%) | | | Mean dwell time (TR) | | | Number of transitions |
| --- | --- | --- | --- | --- | --- | --- | --- |
|  | state1 | state2 | state3 | state1 | state2 | state3 |  |
| Mean difference （TATG - SATG） | -3.078 | 3.906 | -3.073 | -5.002 | 4.350 | 1.019 | 0.069 |
| 95% Confidence interval [Lower Bound, Upper Bound] | -15.964, 9.808 | -9.160, 16.971 | -15.456,9.310 | -25.327, 15.323 | -2.830, 11.531 | -7.332, 9.369 | -1.471, 1.609 |
| *F* | 0.235 | 0.368 | 0.253 | 0.249 | 1.510 | 0.061 | 0.008 |
| *P* | 0.631 | 0.548 | 0.618 | 0.621 | 0.227 | 0.806 | 0.928 |

TATG: true acupuncture treatment group, SATG: sham acupuncture treatment group.

**Figure S.1** The centroid of each functional network connectivity state when the number of clusters k = 4, and the length of sliding time windows W = 20TRs, and the total number of occurrences and percentage of total occurrences are listed above each cluster median.

**Figure S.2** The centroid of each functional network connectivity state when the number of clusters k = 5, and the length of sliding time windows W = 20TRs, and the total number of occurrences and percentage of total occurrences are listed above each cluster median.

**Figure S.3** The centroid of each functional network connectivity state when the number of clusters k = 3, and the length of sliding time windows W = 22TRs, and the total number of occurrences and percentage of total occurrences are listed above each cluster median.

**Figure S.4** The centroid of each functional network connectivity state when the number of clusters k = 3, and the length of sliding time windows W = 30TRs, and the total number of occurrences and percentage of total occurrences are listed above each cluster median.

**Figure S.5** Functional connectivity among distinct states in TATG group and SATG group when the number of clusters k = 4, and the length of sliding time windows W = 20TRs. In circle plots, red lines indicate increased functional connectivity (FC) between or within networks. Blue lines indicate decreased FC between or within networks. SMN(blue block): sensorimotor network. LFPN(green block): left frontoparietal network. RFPN(red block): the right frontoparietal network. DMN(orange block): default mode network. DAN(Purple block):dorsal attention network.

**Figure S.6** Functional connectivity among distinct states in TATG group and SATG group when the number of clusters k = 5, and the length of sliding time windows W = 20TRs. In circle plots, red lines indicate increased functional connectivity (FC) between or within networks. Blue lines indicate decreased FC between or within networks. SMN(blue block): sensorimotor network. LFPN(green block): left frontoparietal network. RFPN(red block): the right frontoparietal network. DMN(orange block): default mode network. DAN(Purple block):dorsal attention network.

**Figure S.7** Functional connectivity among distinct states in TATG group and SATG group when the number of clusters k =3, and the length of sliding time windows W = 22TRs. In circle plots, red lines indicate increased functional connectivity (FC) between or within networks. Blue lines indicate decreased FC between or within networks. SMN(blue block): sensorimotor network. LFPN(green block): left frontoparietal network. RFPN(red block): the right frontoparietal network. DMN(orange block): default mode network. DAN(Purple block):dorsal attention network.

**Figure S.8** Functional connectivity among distinct states in TATG group and SATG group when the number of clusters k =3, and the length of sliding time windows W = 30TRs. In circle plots, red lines indicate increased functional connectivity (FC) between or within networks. Blue lines indicate decreased FC between or within networks. SMN(blue block): sensorimotor network. LFPN(green block): left frontoparietal network. RFPN(red block): the right frontoparietal network. DMN(orange block): default mode network. DAN(Purple block):dorsal attention network.

**Figure S.9** Group difference of FC in brain networks between the TATG group and the SATG group when the number of clusters k =3, and the length of sliding time windows W = 30TRs. In circle plots, red lines indicate increased functional connectivity (FC). SMN(blue block): sensorimotor network. LFPN(green block): left frontoparietal network. RFPN(red block): the right frontoparietal network. DMN(orange block): default mode network. DAN(Purple block):dorsal attention network.

**Figure S.10** Group difference of FC in brain networks between the TATG group and the SATG group when the number of clusters k =3, and the length of sliding time windows W = 30TRs. In circle plots, red lines indicate increased functional connectivity (FC). SMN(blue block): sensorimotor network. LFPN(green block): left frontoparietal network. RFPN(red block): the right frontoparietal network. DMN(orange block): default mode network. DAN(Purple block):dorsal attention network.

**Figure S.11** Group difference of FC in brain networks between the TATG group and the SATG group when the number of clusters k =3, and the length of sliding time windows W = 30TRs. In circle plots, red lines indicate increased functional connectivity (FC). SMN(blue block): sensorimotor network. LFPN(green block): left frontoparietal network. RFPN(red block): the right frontoparietal network. DMN(orange block): default mode network. DAN(Purple block):dorsal attention network.

**Figure S.12** Group difference of FC in brain networks between the TATG group and the SATG group when the number of clusters k =3, and the length of sliding time windows W = 30TRs. In circle plots, red lines indicate increased functional connectivity (FC). SMN(blue block): sensorimotor network. LFPN(green block): left frontoparietal network. RFPN(red block): the right frontoparietal network. DMN(orange block): default mode network. DAN(Purple block):dorsal attention network.
